# Infectious Risk of the Hospital Environment in the Center of Morocco: A Case of Care Unit Surfaces

**DOI:** 10.1155/2020/1318480

**Published:** 2020-05-20

**Authors:** Samira Jaouhar, Abdelhakim El Ouali Lalami, Khadija Ouarrak, Jawad Bouzid, Mohammed Maoulouaa, Khadija Bekhti

**Affiliations:** ^1^Laboratory of Microbial Biotechnology, Faculty of Science and Technology, Sidi Mohammed Ben Abdellah University Fez, Morocco; ^2^Higher Institute of Nursing and Health Professions, Fez, Morocco; ^3^Medical Analysis Laboratory of the Meknes Hospital Center, Regional Health Department Fez-Meknes, Fez, Morocco; ^4^Laboratory of Health Sciences and Technologies, Higher Institute of Health Sciences, Hassan First University, Settat, Morocco

## Abstract

**Background:**

Equipment and hospital surfaces constitute a microbial reservoir that can contaminate hospital users and thus create an infectious risk. The aim of this work, which was carried out for the first time at a hospital in Meknes (regional hospital in the center of Morocco), is to evaluate the microbiological quality of surfaces and equipment in three potential risk areas (burn unit, operating room, and sterilization service).

**Methods:**

This study was carried out over a period of 4 months (February–May 2017). A total of 60 samples were taken by swabbing according to the standard (ISO/DIS 14698-1 (2004)) in an environment of dry area and equipment after biocleaning. Isolation and identification were performed according to conventional bacteriological methods and by microscopic observation for fungi.

**Results:**

The study showed that 40% of surface samples were contaminated after biocleaning. The burn unit recorded a percentage of 70% contamination (*p* value <0.001), 13% for the sterilization service, and 7% for the operating room. 89% of the isolates were identified as Gram-positive bacteria against 11% for fungi (*p* value <0.001). Bacterial identification showed coagulase-negative staphylococci (32%), *Bacillus* spp. (16%), *Corynebacterium* (8%), and oxidase-negative Gram-positive bacillus (40%) while fungal identification showed *Aspergillus niger* (*n* = 2) and *Aspergillus nidulans* (*n* = 1).

**Conclusion:**

To control the infectious risk related to equipment and hospital surfaces, it would be necessary to evaluate the disinfection protocol applied in these units.

## 1. Introduction

Healthcare-associated infections (HAIs) represent a serious public health problem as they lead to increased mortality, morbidity, and costs for patients, their families, and health systems [1, 2]. According to the World Health Organization (WHO), HAIs affect 5 to 10% of patients in developed countries [3]. In developed countries, they affect 25% of hospitalized patients [2]. In the Mediterranean region, the prevalence of HAIs has been 10.5% [1]. In Morocco, the prevalence was 17.8% in a University Hospital (UH) in Rabat (2007) [4], 10.3% (UH Rabat, 2010) [5], and 6.7% (UH Fez, 2007) [6]. In the city of Meknes, a study conducted in 2013 at Mohammed V Hospital revealed that the prevalence of HAI was 9.4% [7]. The microorganisms responsible for HAI were *Escherichia coli*, *Klebsiella pneumoniae*, *Staphylococcus aureus*, and *Pseudomonas aeruginosa* [1, 6]. The services most affected by HAI were the services of surgery, medicine, pediatrics, intensive care, obstetric gynecology, burns unit, and trauma service [7–9]. The factors responsible for HAI are patients (age and immune status), medical practices, and hospital environment (air, surfaces, and water) [10]. The hospital environment is a reservoir of pathogens from patients or the hands of caregivers [11] or the environment [12]. Weber et al. [13] reported that 20% to 40% of HAIs have been attributed to cross-infection via the hands of care staff, which have become contaminated from contact with the patient or by touching contaminated environmental surfaces. The role of the environment in the emergence of HAI has been demonstrated by several studies [14–16]. Thus, it is clear that biomonitoring of the hospital environment is an essential element in the control and prevention of HAI. Biomonitoring provides the information to reduce and prevent the exposure of populations to contaminants in the environment [17]. Microbiological monitoring of the clinical environment, especially surfaces and medical equipment, can be used to detect the presence of nosocomial pathogens and also to evaluate the efficacy of routine cleaning/disinfection practices [18], while the assessments of hospital hygiene indicate that routine cleaning and disinfection may not be performed efficiently and may not be sufficient to eliminate nosocomial pathogens [19, 20]. Many types of nosocomial microorganisms have been found in medical devices and hospital surfaces such as *Clostridium difficile* [21], *Klebsiella pneumoniae* [15, 22], *Staphylococcus aureus* [21, 23], coagulase-negative staphylococci [22–24], and *Acinetobacter baumannii* [13, 14]. So, the aim of our study was (i) to estimate bacterial contamination of medical devices and hospital surfaces and also (ii) to examine the presence of specific nosocomial pathogens in three services with potential risk for patients and care staff (burn unit, operating room, and sterilization service). This investigation was conducted in a hospital with a regional vocation in Meknes (center of Morocco). The results of this study will help the nosocomial infection control committee (NICC) to make a risk analysis strategy to control the risk related to the hospital surface.

## 2. Materials and Methods

### 2.1. Study Site

We carried over a period of 4 months (February–May 2017) the samples from surfaces and medical devices used in three services in a hospital at Meknes (Morocco): burn unit, operating room, and sterilization service. The choice of burn unit was based on the fact that patients hospitalized are immunocompromised and on the result of a study that showed the predominance of clinical bacteria in this service [25]. The choice of other services was based on the French standard (FS S 90 351-2013) [26].

### 2.2. Sampling Technique

The microbiological sampling of the surfaces was carried out by the swabbing technique as recommended by the standard ISO 14698-1 (2004) [27]. Sampling by swabbing technique has been used by several studies that have looked for the presence of nosocomial germs of interest [18]. Cotton swab premoistened in a buffer solution was used for sampling surfaces. At each site, an area of approximately 25 cm^2^ was swabbed and repeated striations in two directions at right angles to each other was carried out in a close zigzag pattern while rotating the swab during sampling of surface ISO 14698-1 (2004) [27]. Then, the swab was wetted in tubes containing Brain Heart Infusion (BHI) broth [28]. The collected samples were transported immediately to the medical laboratory of Mohammed V Hospital and incubated for 24 to 48 h at 37°C. The sampling of each site was repeated three times in order to have a representative result. The Coordination Center of the Committee for the Control of Nosocomial Infections (CCNI) Sud-Ouest (2016) [26] recommends taking samples in these three services, excluding human activity and after biocleaning, except for sterilization where samples taken can be carried out in activity. The selected sampling points are those that may represent a health risk to patients or staff.

### 2.3. Analysis of Samples

Using swabs, we have seeded the suspension on a Petri dish containing the PCA (Plate Count Agar) for the enumeration of microorganisms. Then, the Petri dishes were incubated at 37°C for 24 h to 48 hours. After 24 h of incubation, a number of colonies were noted (the number of colonies was presented in colony-forming unit (CFU)/25 cm^2^).

For isolation, a volume of 10 *μ*L of each sample was inoculated on semiselective media: MacConkey (Enterobacteriaceae), Chapman (staphylococci and micrococci), Cetrimide agar (*Pseudomonas aeruginosa),* and Sabouraud (fungi). The Petri dishes of bacteria were incubated for 24 hours, as for the fungi for 5 to 7 days. The identification of bacteria was carried out according to conventional bacteriology methods. Fungi (molds) were identified by two fundamental examinations: macroscopic and microscopic [29].

### 2.4. Data Processing and Analysis

Data entry was done using Microsoft Office Excel 2010. The descriptive and analytical parts were realized using the XLSTAT extension. To give meaning to the results, we used the chi-square test; the *p* value< 0.001, *p* value <0.01, and *p* value <0.05 were considered highly significant, very significant, and significant, respectively.

## 3. Results

Table 1 shows that autoclave, bedrails, bedside tables, and operating tables are the most contaminated sites with values exceeding the limits of acceptability in the services studied.

**Figure 1:**
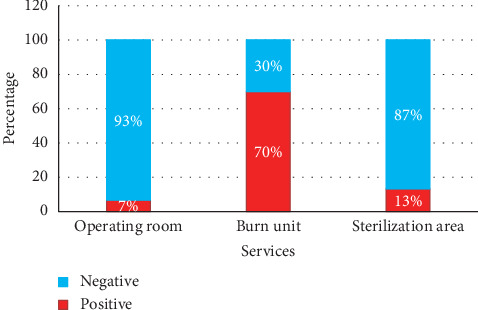
Percentage of contamination by services.

From a total of 60 samples, 40% (*n* = 24) of the samples analyzed were found to be positive and 60% (*n* = 36) were found to be negative. The burn unit (BU) department recorded a very high percentage (70%), followed by the sterilization room (S) with a percentage of 13% and finally the operating room with a percentage of 7% (Figure 1). The distribution of positivity by service is highly significant (chi-squared = 22.639, *df* = 2, *p* value <0.001).

The fungal flora is present in two departments, 67% (*n* = 2) of the molds were found in the BU, whereas only 33% (*n* = 1) of the samples showed the presence of the fungal flora in the sterilization room (SR). A percentage of 89% (*n* = 25) of the isolates were bacteria, while fungi accounted for only 11% (*n* = 3). The distribution of the positive samples according to the germs is highly significant (chi-square = 17.286, *df* = 1, *p* value <0.001). Bacterial identification revealed that all bacteria are Gram-positive.

**Figure 2:**
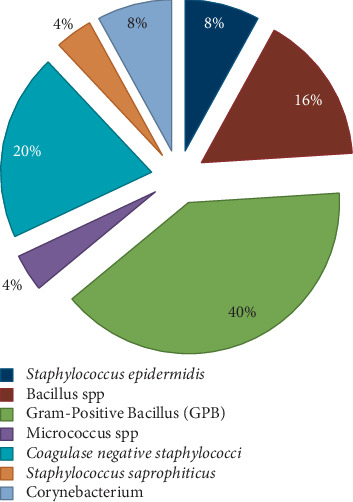
Distribution of isolated bacteria.

They are distributed as follows: 32% (*n* = 8) of Gram-positive cocci were identified as coagulase-negative staphylococci, of which 8% were identified as *Staphylococcus epidermidis* and 4% were *Staphylococcus saprophyticus*, 16 % of bacteria (*n* = 4) were identified as *Bacillus* spp., while *Corynebacterium* spp. were identified with a percentage of 8% (*n* = 2), and finally, 40% of Gram-positive bacillus were unidentifiable (*n* = 10) (Figure 2).

**Figure 3:**
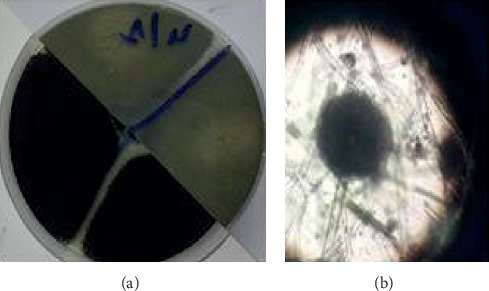
*Aspergillus niger* found in the sterilization service (storage area).

**Figure 4:**
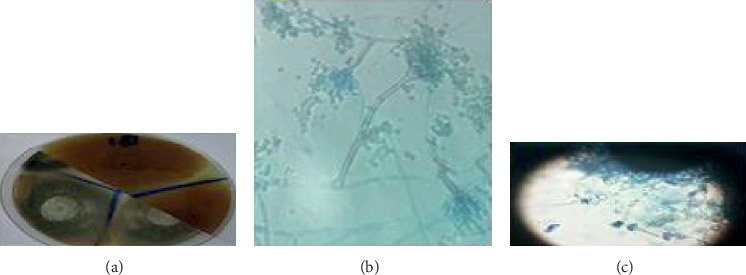
*Aspergillus nidulans* found in burn unit.

The bacterial distribution is statistically very significant (chi-square = 17.28, *df* = 2, *p* value <0.01). From the isolates, only 11 strains have a nosocomial interest: 8 coagulase-negative staphylococci and 3 *Aspergillus* spp. Fungal floras include *Aspergillus niger* (*n* = 2) (Figure 3) and *Aspergillus nidulans* (*n* = 1) (Figure 4).

## 4. Discussion

The hospital environment, especially surfaces, is generally colonized by many opportunistic and pathogenic microorganisms. The surfaces are contaminated by microorganisms derived from the patient himself or sedimentation of particles in the air [30]. Several pathogens can survive for days and months in dry surface and also can be considered as the source of HAI [31]. The role of the environment in the development of HAI was poorly documented except for environmental microorganisms (Aspergillosis, Legionellosis) [32].

Nevertheless, studies have shown this relationship [14, 15]. Microbiological surface checks are necessary to prevent and limit the transmission of microbiological risks between the environment and humans. Our study showed that 40% of the samples were found positive. The burn unit was the most concerned area with very high contamination (70%), followed by the sterilization service (13%) and only 7% for the operating room. A study conducted at a hospital in Fez (Morocco) reported a preponderance of bacterial strains as an emergency operating room and operating room [24]. Moreover, the contamination of the operating room has also been demonstrated by Saouide el Ayne et al. [33]. The most contaminated sites were autoclave, bedrails, bedside tables, and operating tables. Several studies have shown that the most contaminated sites are those that are in frequent contact with caregivers and patients [34, 35]. Meunier et al. also reported and observed severe contamination of hospital surfaces where they found that the medical devices and surface close to patients are largely covered by pathogen microorganisms [28].

The total microorganisms count in the sterilization service showed that the acceptability limits were exceeded (CFU/25 cm^2^ > 30) [26]. We also found that the limit values were exceeded in the patient's room (burn unit) and the operating room (CFU/25 m^2^ >10) according to the Association for the Prevention and Contamination Study (APCS).

In this study, bacteria represent 89% of isolates against 11% for fungi. Several studies have reported contamination of the hospital environment with fungi [36, 37]. Identification of fungi revealed the presence of *Aspergillus niger* (sterilization service and patient's room) and *Aspergillus nidulans* (patient's room). This result requires a review of hygiene procedures in these services [26]. Infections due to *Aspergillus* spp. result in significant mortality and morbidity. *Aspergillus niger* has been associated with pulmonary disease [38], cutaneous infections [39], and otomycosis [40].

Our result showed the presence of only Gram-positive bacteria. Other studies have shown the presence of Gram-positive bacteria and Gram-negative bacteria [24, 37, 41]. Another study has shown that Gram-positive bacteria can survive in dry environments for longer periods than Gram-negative bacteria [42]. This result could be explained by the desiccation resistance of Gram-positive versus Gram-negative bacteria [43]. Bacterial identification determined coagulase-negative staphylococci at 32%, *Bacillus* spp. at 16%, and *Corynebacterium* spp. at 8%. The medical devices of the Iranian hospitals were colonized by coagulase-negative staphylococci [22]. The results of our study are consistent with those found in El Idrissi Hospital in Kenitra [33]. Surveillance of the microbiological quality of surfaces and equipment carried out at two hospitals in Morocco showed the presence of coagulase-negative staphylococci [14] and *Bacillus* spp. [24, 37]. Meunier et al. reported that *Bacillus* spp. bacteria are constantly present in the environment and do not appear to be accessible to biocleaning, and this could be explained by their ability to sporulate [28]. The presence of these bacteria in the patient's immediate environment can cause infectious diseases. Indeed, coagulase-negative staphylococci are recognized as a cause of nosocomial infections [44] including endocarditic for immunocompromised patients [45]. Microorganisms found on surfaces depend on several factors: (i) human activity, which results in a supply of microorganisms by patients and staff [46], and (ii) the quality of biocleaning and characteristics of microorganisms (adhesion to inert surfaces, ability to produce a biofilm, etc.) [47]. High contamination and the presence of microorganisms of human and environmental origin in medical devices and surfaces could be linked, on the one hand, to inefficiency disinfectants and, on the other hand, poor hygiene and inappropriate application cleaning procedures.

The results of our study showed that the surfaces and medical devices of the services studied are colonized by pathogens that could cause HAI. These infections can be reduced by appropriate hand hygiene [48] and appropriate disinfection of surfaces and medical devices [2]. In recent years, a number of studies have demonstrated that environmental disinfection and cleaning interventions can reduce HAI [49, 50]. Notably, Datta et al. [51] and Grabsch et al. [52] demonstrated that methicillin-resistant *Staphylococcus aureus* acquisition and vancomycin-resistant *Enterococcus* were reduced by disinfection intervention to the rooms previously occupied by patients colonized by the same pathogen. This study raises the question of the effectiveness of the disinfectants used in these services.

## 5. Conclusion

This study made it possible to know the microbial ecology of the three services at potential risk (burn unit, operating room, and sterilization service). The identification revealed the exclusive presence of Gram-positive bacteria with a preponderance of coagulase-negative staphylococci and the presence of fungal agents of *Aspergillus niger* and *Aspergillus nidulans*. To control the infectious risk related to the environment hospital, especially the surfaces, it would be necessary to evaluate the disinfection protocol applied to the surfaces.

## Figures and Tables

**Table 1:** CFU/25 cm^2^ found in the different critical points of the three services studied.

	Services	Critical points	CFU/25 cm^2^ (*M* ± *σ*)
Burn unit	Operating room	Operating table	0
		Instruments table	0
		Anesthesia mask	0
	Patient's room 1	Cart	3 ± 1.67
		Bed rails	5 ± 4.67
		Bedside tables	8 ± 6.67
		Refrigerator	7 ± 4.33
	Patient's room 2	Cart	41 ± 25.33
		Bedside tables	87 ± 47.33
		Bed rails	173 ± 100
Operating room	Operating table	58 ± 33.33	
	Instruments table	0	
	Anesthesia table	0	
	Mask of anesthesia	0	
	Scialytic	0	
Sterilization	Conditioning area	Paillasse	0
		Autoclave	115 ± 66.67
	Storage area	Cart	0
		Paillasse (1)	0
		Paillasse (2)	1 ± 0,.3

*M* ± *σ*: mean ± standard deviations. CFU: colony-forming unit.

## Data Availability

Data used to support the findings of this study are included within the article and also available from the corresponding author upon request.
